# 2D data arrangement to train ANN for depression levels measurement

**DOI:** 10.1016/j.dib.2025.111429

**Published:** 2025-02-26

**Authors:** Al Fathjri Wisesa, Eny Latifah, Sutrisno, Suyatno, Tutut Chusniyah, Kukuh Setyo Pambudi, Mochamad Khoirul Rifai, Moh. Fariq Firdaus Karim, Anugerah Agung Dwi Putra

**Affiliations:** a Department of Physics, Faculty of Mathematics and Natural Sciences, State University of Malang, Jl. Semarang 5, Malang, 65145, Indonesia; b Department of Physics, Faculty of Science and Data Analytics, Institut Teknologi Sepuluh Nopember (ITS), ITS Campus, Surabaya, 60111, Indonesia; c Department of Psychology, Faculty of Psychology, State University of Malang, Jl. Semarang 5, Malang, 65145, Indonesia

**Keywords:** Artificial Neural Network (ANNs), Physical parameters, Psychological parameters, 2D Data, Train ANNs

## Abstract

We arranged data to train Artificial Neural Networks (ANNs) designed as a depression-level measurement tool. Even though, as an advanced form of stress, depression impacts many physical parameters disorder, measuring depression using only physical parameters is insufficient. It is urgent to integrate comprehensively psychological and physical parameters as two dimensions, 2D, data. We harvested the dataset of 95 respondents from college students. The physical dimension consisted of four parameters measured noninvasively, and the psychological dimension was assessed using the Perceived Stress Scale (PSS). The initial analysis revealed notable correlations between increased stress perception and certain physical parameters analysis, particularly an elevated heart rate and reduced sleep quality. The highly significant p-value provided strong evidence that the observed difference in means is not coincidental. According to data processing, we have the data set including all levels of depression to enhance the effectiveness of measuring depression. Using two-dimensional data, we aim for the ANNs to learn interaction patterns between these parameters, improving accuracy in depression detection.

Specifications Table

**Table:** 

Subject	Artificial Intelligence; Instrumentation
Specific subject area	Measurement tools of depression levels using Artificial Neural Networks that need to be learned by integration of physical and psychological dimensions
Type of data	Graphs, Figures, and Tables
Data collection	Physical Parameters: • Heart Rate (BPM): Measured twice using a pulse oximeter, repeat data collection with a certain number (n > 5) • Oxygen Saturation (SpO2): Measured twice using a pulse oximeter, repeat data collection with a certain number (n > 5) • Body Temperature: Assessed using a non-contact infrared thermometer at the end of the data collection phase • Sleep Duration: Respondents self-reported their average sleep duration over the course of one week Psychological Parameters: • Perceived Stress Scale (PSS): Psychological stress was assessed through psychological questionnaire, which was completed by all respondents
Data source location	Department of Physics, Faculty of Mathematics and Natural Sciences, State University of Malang, Malang, Indonesia
Data accessibility	Repository name: Mendeley Data Data identification number: 10.17632/x3tzxg38wj.5 Direct URL to data: https://data.mendeley.com/datasets/x3tzxg38wj/5

## 1 Value of the Data

• **Global Trends and Resource Optimization:** The collected student mental health data highlights significant trends in mental health monitoring, underscoring the importance of obtaining accurate and comprehensive datasets. These insights support efforts in optimizing resources and enhancing the provision of effective mental health support within academic environments.
• **Valuable Insights from the Dataset:** The dataset encompasses both physical parameters (heart rate, oxygen saturation, body temperature, and sleep duration) and psychological parameters (stress, anxiety, depression, and emotional health use the PSS questionnaire). This dual nature of the data offers valuable support for the development of mental health strategies, aiding in decision-making processes and in predicting the prevalence of depressive disorders among college students based on the interplay between physical and psychological parameters.
• **Suitability for Machine Learning:** This dataset is well-positioned for machine learning applications. Preliminary analysis indicates that scaling and normalization techniques have been applied, though they have not yet demonstrated significant improvements in outcomes. Nonetheless, the dataset presents a promising opportunity for the implementation of advanced predictive models and analytics.
• **Integrated Perspective on Mental Health Effects:** By incorporating both physical and psychological data, decision-makers can obtain a comprehensive view of the effects of mental health issues among college students. The interactive visualization tools provided allow for integrative analysis, enhancing the understanding of mental health impacts.
• **Coding, Replicability, and Flexibility:** The dataset is accompanied by code that facilitates replication, validation, and visualization of the analysis. This flexible code allows researchers to further analyze this dataset or explore additional datasets for future studies.

## 2 Background

Depression can develop from stress, defined as mental or emotional strain caused by unavoidable demands [1]. Prolonged exposure to stress disrupts relationships, work, health, and well-being, often leading to emotional disturbances [2]. Accurate data allows healthcare providers to assess depression prevalence across age and cultural groups, guiding targeted interventions [3]. Depression is influenced by genetic, biological, and psycho-social factors [4]. Its physical symptoms are connected to neurotransmitters which regulate mood and pain, linking physical and mental health indicators [5].

Relevant studies that inform this research include the works of Rachakonda et al. [1] and Harris et al. [8], which adopt distinct approaches to assessing depression levels. Rachakonda et al. [1] emphasized physical parameters, developing a method to analyze physiological changes during sleep, such as heart rate, brain wave activity, and other biomarkers. Their methodology incorporates machine learning algorithms to predict stress levels and offer stress management solutions. However, this study overlooks the role of psychological parameters as potential predictors in depression assessment.

Conversely, Harris et al. [8] centered their research on psychological parameters, employing the Perceived Stress Scale (PSS) to measure depression levels without integrating physical data. While their findings demonstrated improved accuracy compared to earlier studies, their analysis lacked depth in considering participants' physical conditions. This limitation raises questions about the extent to which physical factors might influence depression measurements.

In mental health research, depression cannot be fully encapsulated by either physical or psychological parameters alone. An integrative approach that combines both dimensions is essential to provide a more holistic understanding. Exploring the interplay between physical and psychological factors has the potential to yield novel insights and extend beyond traditional paradigms in mental health studies.

Currently, there is no dataset explicitly designed to explore the integrative relationship between physical and psychological parameters in depression measurement. This research seeks to address this gap by employing an artificial neural network (ANN), enabling sophisticated data processing to uncover correlations and the dynamic interactions between these parameters in influencing depression levels.

ANNs is a powerful tool for complex system analysis, widely applied in healthcare for diagnosis, analysis, and prediction [[6], [7], [8], [9]], as an effective depression measurement by analyzing Gaussian-distributed data, which reflects natural variation in mental health, leading to more reliable results. The accuracy enhancement of the ANNs involves applying machine learning for complex data pattern processing and the quality of predictions [10].

Depression is more common among young individuals, with higher prevalence in gender [11], increasing among college students [[12], [13]] including stress, anxiety, and depression [14]. Providing a depression level measurement using ANNs is urgent. For more accuracy, both physical and psychological dimensions must be trained to ANNs as a comprehensive view of depression.

## 3 Data Description

The dataset presented in this submission encompasses the results of a comprehensive survey conducted among students during the study period. The survey gathered both physical and psychological variables, including heart rate, oxygen saturation, body temperature, and sleep duration, along with psychological stress levels measured using the psychological questionnaire. The submission includes a Python Jupyter notebook containing code for data processing, visualization, and machine learning-based analysis, aimed at predicting depression levels from the collected physical and psychological metrics. The primary objective of this analysis is to identify patterns and relationships between these variables, offering valuable insights that can inform decisions regarding students’ mental health and well-being.

### 3.1 Physical data

The dataset contains critical health variables, including heart rate, oxygen levels, body temperature, and sleep duration, all of which have potential associations with depression [15].

• **Heart Rate:** There is a documented relationship between depression and heart rate. Depression, particularly Major Depressive Disorder (MDD), has been linked to an increased incidence of cardiovascular diseases. This highlights the importance of monitoring heart rate in patients with depressive symptoms to understand potential cardiovascular risks [3].
• **Blood Oxygen Levels:** Stress and anxiety can impact respiratory function, which in turn can alter blood oxygen levels. Early research indicates that stress and anxiety may reduce blood oxygen levels during medical procedures such as periodontal surgery, making it important to monitor oxygen saturation in individuals with mental health disorders [4].
• **Body Temperature:** Studies have shown a connection between depression and body temperature regulation. For instance, elevating body temperature to the level of a mild fever in individuals with depression has been found to exacerbate depressive symptoms [5].
• **Sleep Duration:** According to the Diagnostic and Statistical Manual of Mental Disorders-IV (DSM-IV), insomnia, defined by difficulties in initiating or maintaining sleep, or waking up prematurely, is a common symptom of depression. This underscores the role of sleep disturbances in mental health assessment and treatment [6].

Addressing depression is critical due to its profound impact on both physical and mental well-being. It can worsen underlying physical conditions, increase social isolation, reduce treatment adherence, and elevate the risks of despair and premature mortality. Effective treatment approaches include antidepressant medications and psychological therapies such as cognitive and interpersonal therapy [7].

### 3.2 Psychological data

Psychological data were gathered using the PSS questionnaire, a widely used tool for assessing an individual's perception of stress. Developed by Sheldon Cohen in 1983, the PSS measures how stressful individuals perceive their life situations to be [8]. The PSS questionnaire consists of 10 items, each rated on a 5-point Likert scale (0 = Never, 4 = Frequently). Respondents evaluate the frequency of specific feelings and thoughts experienced over the past month [9]. The later items in the PSS focus on how much control, anxiety, or overwhelming feelings a person experiences in daily life. Higher PSS scores indicate a higher level of perceived stress [10].

### 3.3 Dataset documentation

The sample criteria for the dataset have been carefully aligned with the characteristics of the target population, specifically undergraduate students. The dataset includes individuals aged 18 years or older who are pursuing undergraduate or equivalent education. This criterion reflects findings from previous studies by [12] and [13], which highlight an increasing prevalence of depression among students.

The dataset comprises both physical and psychological parameters collected from students over a single day at Universitas Negeri Malang. The physical variables include heart rate, oxygen saturation, body temperature, and sleep time, while the psychological variables are measured using the psychological questionnaire based on Perceived Stress Scale (PSS) on December 7, 2023. The dataset comprises a sample of 95 individuals, collected through a comprehensive questionnaire encompassing both physical and psychological parameters as two dimensionals (2D) of depression component. The physical parameters measured, as a physical dimension, include body temperature, heart rate, oxygen saturation, and sleep duration, providing a detailed overview of respondents’ physical conditions. Psychological parameters, as a psychological dimension, are focused on assessing mental well-being, with emphasis on stress, anxiety, depression, and emotional health. The psychological questionnaire is employed to quantify stress levels and evaluate the overall psychological state of the respondents Table 1.

**Table 1:** Description of variables contained in the dataset provided.

Variable Name	Description
Nama	Student name or unique identification code for each study respondent
BPM	Average heart rate of students was measured when filling out the psychological questionnaire
SPO2	Percentage of oxygen saturation (SpO_2_) in blood was measured when filling out the psychological questionnaire
Suhu Tubuh	Student body temperature (°C) was measured when filling out the psychological questionnaire
Waktu Tidur	Student answer to the amount of sleep (hours) during the past week
Psikologi 1	Student answer to question 1 of the psychological questionnaire
Psikologi 2	Student answer to question 2 of the psychological questionnaire
Psikologi 3	Student answer to question 3 of the psychological questionnaire
Psikologi 4	Student answer to question 4 of the psychological questionnaire
Psikologi 5	Student answer to question 5 of the psychological questionnaire
Psikologi 6	Student answer to question 6 of the psychological questionnaire
Psikologi 7	Student answer to question 7 of the psychological questionnaire
Psikologi 8	Student answer to question 8 of the psychological questionnaire
Psikologi 9	Student answer to question 9 of the psychological questionnaire
Psikologi 10	Student answer to question 10 of the psychological questionnaire
Level Depresi Berdasarkan Parameter Fisis	Depression level categories based on results from physical variables
Level Depresi Berdasarkan Parameter Psikis	Depression level categories based on results from psychological variables
Level Depresi	Depression level categories from a combination of physical and psychological variables

The psychological questionnaire is widely regarded as one of the most frequently employed instruments for assessing an individual's subjective perception of stress. Originally developed by Sheldon Cohen in 1983, the PSS was designed to evaluate the degree to which various situations in a person's life are appraised as stressful [9]. Rather than focusing solely on discrete or identifiable stress-inducing events, the PSS takes a broader approach by capturing how individuals cognitively appraise the demands they encounter in daily life and their perceived ability to cope with these challenges [10].

### 3.4 Structure of information

**Fig. 1:**
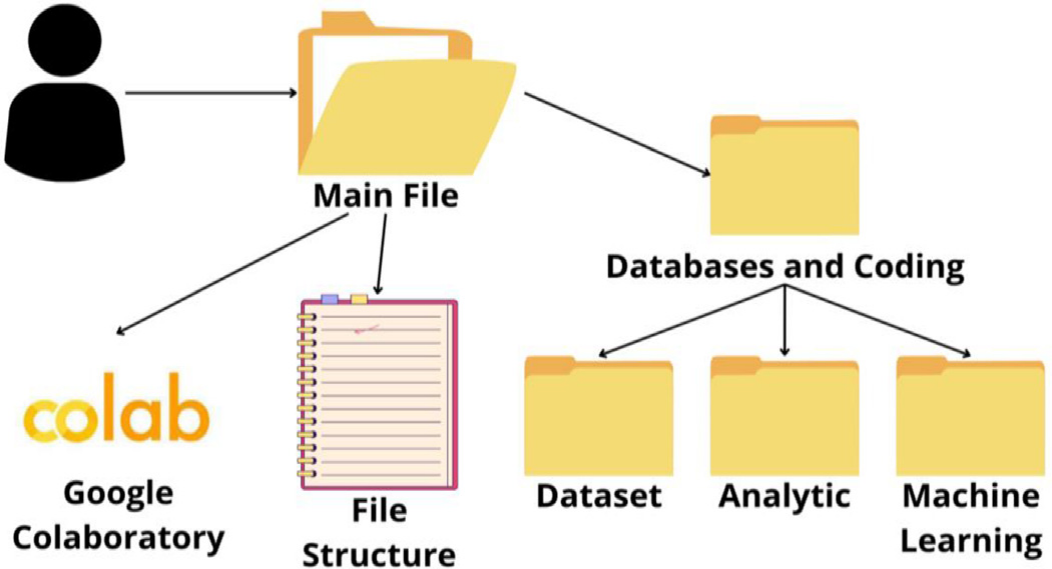
Structure of the information.

The data description and analysis are facilitated through two interactive platforms: Jupyter Notebook serves as the environment for analysis, while Python handles the data processin. Both are designed to enhance the reader's understanding of the dataset. Furthermore, a ``File Structure'' document is included, serving as a guide to the file organization and offering concise descriptions of each file's purpose and usage (Fig. 1). All datasets are stored within the “Databases and coding/Databases” subfolder. This structure facilitates a smooth integration of data analysis, which is carried out using Python within the Jupyter Notebook.

### 3.5 Google collab

The Google Colaboratory notebook titled “Depression_Analysis_Psychology_Physics.ipynb”, accessible via the **Mendeley Data repository** [16], is developed to explore the interplay between psychological and physical variables in college students. Specifically, it investigates the relationship between psychological dimensions (psychological questionnaire) and physical dimensions (heart rate, oxygen saturation, body temperature, and sleep duration).

The notebook begans by outlining the objective of the analysis, focusing on evaluating how these two dimensions contribute to depression levels among college students. It also provides references to the Python scripts employed for data analysis and visualization. The initial phase of data processing involves cleaning and preparing the dataset, followed by presenting descriptive statistics for both two dimensions.

In the core relationship analysis, the notebook calculates and visualizes correlations between the two dimensions, along with examining data distributions and identifying patterns in these relationships. Graphical tools, such as plots and graphs, are used to elucidate the connections between the variables, enhancing interpretability.

This data can be utilized to train machine learning models, enabling the identification of key factors influencing depression and facilitating accurate predictions based on the available information. The machine learning component of the notebook involves preprocessing the data and applying algorithms such as regression or clustering to detect patterns and make predictive assessments about depression levels.

The notebook concludes with a discussion of the results, summarizing key findings and drawing conclusions regarding the relationships between two dimensions. As a collaborative tool, the notebook offers a thorough approach to understanding how these variables interact and contribute to depression levels in students.

## 4 Experimental Design, Materials and Methods

### 4.1 Experimental design

This study focuses on examining the relationship between physical and psychological parameters to detect depression levels in individual students during a specific period. The research employs a cross-sectional design, in which data is collected at a single point in time from a sample of 95 students who participated in the study. Additionally, the study seeks to explore how these physical and psychological factors may contribute to or influence the onset and severity of depression.

Despite incorporating 95 final respondents, it is acknowledged that the sample size may not fully represent the broader student population. This limitation could influence the statistical power of the analysis and reduce the generalizability of the findings. Key challenges include limited funding to expand the scope of data collection and a low level of respondent participation. These and other obstacles encountered during the data collection process are comprehensively addressed in the LIMITATIONS section.

### 4.2 Protocol description

**Fig. 2:**
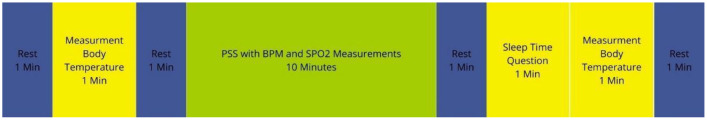
Data acquisition protocol. The blue blocks represent the rest segments, the yellow and green ones report the tasks including their duration.

To ensure a robust and integrated approach to data collection, an extensive literature review [3-[4], [5], [6]] was conducted to combine physical and psychological parameters, aiming to derive more accurate and realistic assessments of depression. Given that cognitive states are easily influenced in controlled laboratory settings and typically activated in conscious contexts, a tailored data collection protocol was developed, as illustrated in Fig. 2. The scientific methodology of this protocol has been rigorously validated by the Research Ethics Committee of Malang State University (Approval Number: 27.9.10/UN32.14/PB/2024).

The data acquisition begins with a 1-minute rest period to ensure the individual is in a calm state before any measurements are taken. Following this, the individual's body temperature is measured and recorded, a process that takes 1 minute. After this initial measurement, a second 1-minute rest period is observed to minimize any external stress factors that might affect subsequent readings. Next, a 10-minute session is conducted where the individual undergoes a psychological questionnaire, while their BPM (beats per minute) and SpO_2_ (oxygen saturation) levels are simultaneously measured using appropriate monitoring devices. Once this 10-minute assessment is completed, the individual is given another 1-minute rest period.

After the rest, the individual is asked a question related to their sleep time, which takes 1 minute to complete. This could involve querying their sleep duration, quality, or other relevant sleep patterns. Following the sleep question, the body temperature is measured again to track any changes from the initial reading, which also takes 1 minute. Finally, the procedure concludes with a final 1-minute rest period, allowing the individual to relax and recover from the series of measurements and assessments.

### 4.3 Materials

The materials used in this study were carefully selected to ensure precise data collection for both physical and psychological dimensions. The instruments employed include:

• **Psychological Questionnaire:** This tool is designed to measure an individual's perceived level of stress over a given period. The psychological questionnaire consists of 10 items rated on a Likert scale ranging from 0 to 4, where respondents assess how frequently they have encountered symptoms of stress in the preceding weeks.
• **Physical Measuring Instruments:**
  a. Digital Thermometer: Used to measure participants’ body temperature.
  b. Heart Rate Monitor (BPM): Records the average heart rate of participants during the study.
  c. Pulse Oximeter: Measures the oxygen saturation (O2) in the participants’ blood.
  d. Sleep Tracker: Monitors the duration and quality of participants’ sleep throughout the study.
• **Software:**
  a. SPSS: Employed for statistical analysis and data processing. SPSS was instrumental in calculating descriptive statistics, correlations, and conducting other statistical tests relevant to the study.
  b. Python: Utilized for more advanced data analysis, particularly in developing machine learning-based predictive models and performing multivariate analyses to explore deeper patterns within the data.

### 4.4 Research variables

In this study, the independent variables include both physical and psychological parameters. The physical parameters consist of body temperature, heart rate, oxygen saturation, and sleep duration, while the psychological parameters encompass stress, anxiety, depression, and emotional well-being, as measured by the psychological questionnaire. The dependent variable is the score representing the level of depression. Control variables include demographic factors such as age and academic semester, which are considered potential confounders that could influence the relationship between the physical and psychological parameters and depression levels. By controlling for these demographic variables, the study aims to isolate the effects of the independent variables on depression.

### 4.5 Methods

The data collection procedure for this study was divided into two key components: completing the psychology questionnaire and measuring physical parameters using calibrated devices. Data collection spanned several days to capture representative physical data. To minimize variability due to timing, participants were instructed to take measurements at the same time each day.

• **Questionnaire:** Respondents completed the psychological questionnaire online, using either a mobile device or computer. The completion process was supervised by the researchers to ensure that all items were answered correctly and consistently.
• **Physical Measurements:** The physical measurements, including body temperature, heart rate, oxygen saturation, and sleep time, were taken using calibrated devices. Body temperature was measured daily at a consistent time, while heart rate and oxygen saturation were recorded in a resting state. These measurements aimed to capture accurate and stable readings of the participants’ physical dimensions.

### 4.6 Data calculations

The classification of physical parameter values is determined based on the following predefined groups in Table 2.

**Table 2:** Points of each physical parameter.

Points	Heart Rate (bpm)	Oxigen Level (%)	Body Temperature (°C)	Sleep Duration (Hours)
1	< 60	< 90	< 34	< 2
2	60 – 65	90 – 92	34 – 36	2 – 5
3	> 65	> 92	> 36	> 5

The final score for “Level Depresi Berdasarkan Parameter Fisis” is obtained by averaging the points assigned to each physical parameter (heart rate, oxygen level, body temperature, and sleep duration). Similarly, the “Level Depresi Berdasarkan Parameter Psikis” is derived from the total points accumulated from the respondent's answers to the psychological questionnaire, with each question weighted appropriately.

The integrated classification of depression levels based on physical and psychological parameters is determined using the Table 3.

**Table 3:** Determine of parameter level.

Total Point of Physical Parameter	Total Point of Psycological Parameter	Parameter Level
1–4	0–13	1 (Low)
5–8	14–26	2 (Moderate)
9–12	27–40	3 (High)

The overall of “Level Depresi” is calculated by summing the average levels of physical parameters (1–3) and psychological parameters (1–3). This integrative approach provides a more comprehensive assessment of the respondent's depression level by combining both physical and psychological indicators.

### 4.7 Analysis of descriptive statistic

Table 4 provides a summary of descriptive statistics for the respondents’ physical parameters, including heart rate, oxygen saturation, body temperature, and sleep duration. The statistics encompass sample size, mean, standard deviation, as well as the minimum, quartile (25^th^, 50^th^, 75^th^ percentiles), and maximum values.

**Table 4:** Respondents' physical parameters.

	Heart Rate	Oxygen Saturation	Body Temperature	Sleep Duration
Count	95	95	95	95
Mean	99.14	93.52	36.31	5.847
Standard Deviation	11.34	10.77	0.698	1.331
Minimum	64.60	93.80	34.00	8.00
25%	91.10	93.40	35.90	5.00
50%	100.0	96.20	36.50	6.00
75 %	107.5	97.60	36.77	7.00
Maximum	122.2	99.40	37.90	9.00

Table 5 presents the descriptive statistics for psychological variables assessed using the psychological questionnaire. This includes data for all ten psychological questionnaire items, reporting the sample size, mean, standard deviation, and the corresponding minimum, quartiles, and maximum values.

**Table 5:** Respondent psychological parameters.

	P1	P2	P3	P4	P5	P6	P7	P8	P9	P10
Count	95	95	95	95	95	95	95	95	95	95
Mean	2.3	1.7	2.1	1.5	2.0	1.7	1.8	2.7	1.9	1.8
Standard Deviation	0.91	1.05	0.99	0.76	0.79	0.85	0.95	0.94	1.03	0.93
Minimal	1.0	0.0	0.0	0.0	0.0	0.0	0.0	1.0	0.0	0.0
25 %	2.0	1.0	1.0	1.0	1.0	1.0	1.0	2.0	1.0	1.0
50%	2.0	2.0	2.0	1.0	2.0	2.0	2.0	3.0	2.0	2.0
75 %	3.0	2.5	3.0	2.0	2.0	2.0	2.5	3.0	3.0	2.0
Maximal	4.0	4.0	4.0	4.0	4.0	4.0	4.0	4.0	4.0	4.0

Table 6 summarizes the respondents’ depression levels, categorized based on physical factors, psychological factors, and their combination. The statistical data include the sample size, mean, standard deviation, and the range of values, described through the minimum, quartiles, and maximum for each criterion.

**Table 6:** Respondents' depression level.

	Physical	Psychological	Combined
Count	95.00	95.00	95.00
Mean	1.557	1.894	1.968
Standard Deviation	0.540	0.494	0.307
Minimal	1.00	1.00	1.00
25 %	1.00	2.00	2.00
50%	2.00	2.00	2.00
75 %	2.00	2.00	2.00
Maximal	3.00	3.00	3.00

### 4.8 Analysis of inferencial

• **T-Test**
  The t-test is a statistical method employed to evaluate whether the means of two groups or variables differ significantly. In this study, the t-test was utilized to compare the mean values of physiological and psychological parameters measured in participants, aiming to determine whether there is a statistically significant difference between these groups, as shown in Table 7. This test plays a critical role in assessing hypotheses concerning the differences between variables under study.

**Table 7:** The t-test results of physical and psychological parameters.

Statistical Indicator	Value
T-statistic	-4.363253
Degrees of freedom	94
P-value	0.000033
Mean difference	-0.336842
Standard error	0.07511
  The t-test results revealed a significant difference in the mean values of the physiological and psychological parameters. With a t-statistic of -4.363253 and a very small p-value (0.000033), the results allow for the rejection of the null hypothesis, which posited no difference in means between the two groups. The highly significant p-value indicates strong evidence that the difference in means is not due to chance.
  The mean difference of -0.336842 signifies that the average value of the physiological parameters is statistically different from the psychological parameters, with the difference expressed in terms of standard deviation units. This suggests a meaningful relationship between physiological and psychological variables in this study, offering insights into how physiological factors may influence or correlate with mental health and stress.

• **ANOVA (Analysis of Variance) Test**
  The ANOVA test is a statistical method used to compare the means of three or more groups or variables to assess whether there are significant differences among them. In this study, ANOVA was applied to both physiological and psychological parameters to evaluate whether significant differences in mean values exist between the groups tested.
  The results of the ANOVA test for physical parameters (Table 8), indicate no statistically significant differences between the groups. Although some parameters exhibited F-statistic values that approached significance, the high p-values suggest that the observed differences are not large enough to be considered significant at the conventional alpha level (typically 0.05). This implies that the variability in physical parameters across the groups is insufficient to support a clear statistical distinction.

**Table 8:** ANOVA test for physical parameters.

	Sum Square 1	Sum Square 2	DF1	DF2	Mean Square 1	Mean Square 2	F Stat	P Value
°C	1.431	45.56	2	92	0.715	0.495	0.267	0767
BPM	3241	11437	2	92	1620	134.5	2.69	0.063
SpO2	1315	10480	2	92	657.9	113.9	1.89	0.156
Time Sleep	15.33	163.6	2	92	7.66	1.779	0.80	0.452
  Similarly, the ANOVA results for the psychological parameters for P1 through P10, as shown in Table 9, show no significant differences in mean values between the groups. The consistently high p-values for all psychological parameters indicate that the observed variations are likely due to random chance, rather than meaningful differences, and are not statistically significant at the alpha level of 0.05. Therefore, the differences in psychological parameters measured in this study do not support the hypothesis of significant group differences.

**Table 9:** ANOVA test for psychological parameters.

	Sum Square 1	Sum Square 2	DF1	DF2	Mean Square 1	Mean Square 2	F Stat	P Value
P1	0.002	78.88	2	92	0.001	0.857	0.000	0.999
P2	1.200	105.2	2	92	0.600	1.143	0.098	0.905
P3	2.999	92.17	2	92	1.496	1.001	0.881	0.417
P4	5.993	53.24	2	92	2.996	0.578	1.504	0.227
P5	1.728	53.28	2	92	0.864	0.644	0.553	0.576
P6	0.812	69.06	2	92	0.406	0.750	0.173	0.841
P7	2.664	83.63	2	92	1.332	0.909	0.916	0.403
P8	0.614	84.18	2	92	0.407	0.915	0.130	0.877
P9	0.047	100.8	2	92	0.023	1.096	0.004	0.995
P10	0.42	82.53	2	92	0.214	0.897	0.053	0.947

## Limitations

• **Cross-Sectional Research Design.** Data were collected at a single point in time, which limits the ability to establish cause-and-effect relationships between physical and psychological dimensions. Longitudinal studies would be better suited to capture changes over time.
• **Limited Sample Size.** With 95 respondents, the sample size may not be fully representative of the broader population. A larger sample could improve the statistical power of the analysis and enhance the generalizability of findings.
• **Self-Report in Psychological Measurement.** The reliance on self-reported data for psychological parameters introduces subjectivity and potential recall bias, which could affect response reliability. Adding qualitative methods could help mitigate this bias.
• **Physical Measurement Constraints.** Physical parameters were assessed under limited conditions. Factors like physical activity, diet, and daily routines were not consistently controlled, possibly impacting measurement accuracy.
• **Influence of External Factors.** Key variables such as socioeconomic status and work environment, which could affect stress and physical health, were not directly measured, limiting a comprehensive view of factors influencing mental health.
• **Generalizability to a Wider Population.** The study's sample may lack social, economic, and cultural diversity, reducing the applicability of the findings to broader populations.

## Ethics Statement

This study was conducted with the utmost consideration for ethical standards in research involving human participants. All respondents voluntarily participated in the research, and informed consent was obtained prior to data collection. Participants were fully informed about the purpose of the study, the nature of their participation, and the handling of their data. All personal information was anonymized to ensure confidentiality, and no identifying details were included in the analysis or reporting of the results.

The physical and psychological dimensionals collected, including heart rate and perceived stress levels, were measured non-invasively and with participants’ full knowledge and consent. In cases where participants exhibited signs of significant distress or depression, appropriate support services were recommended to ensure their well-being.

The study followed ethical guidelines as outlined by **Universitas Negeri Malang review boards (Number 27.9.10/UN32.14/PB/2024)** and adhered to local regulations governing research with human subjects in Indonesia. Furthermore, the research team remained sensitive to the cultural context, ensuring that the approach was respectful of the participants’ backgrounds and aligned with best practices in mental health research.

The integration of machine learning and predictive modeling was done with an emphasis on the responsible use of data, ensuring that the analysis focused on improving student well-being rather than stigmatizing individuals with mental health concerns. This study aims to contribute to the development of more effective mental health strategies within academic settings, and care was taken to ensure that its findings are applied in ways that support students rather than exacerbate their challenges.

## Credit Author Statement

**Al Fathjri Wisesa:** Conceptualization, Methodology, Data Curation, Writing (Original Draft Preparation); **Eny Latifah:** Supervision, Validation, Writing (Review & Editing); **Sutrisno:** Formal Analysis, Writing (Review & Editing); **Suyatno:** Investigation, Resources, Project Administration; **Tutut Chusniah:** Data Curation, Statistical Analysis, Psychological Assessment Validation; **Kukuh Setyo Pambudi:** Data Curation, Statistical Analysis, Psychological Assessment Validation; **Mochamad Khoirul Rifai:** Supervision, Project Administration, Funding Acquisition, Writing (Review & Editing); **Moh. Fariq Firdaus Karim:** Data Curation, Statistical Analysis; **Anugerah Agung Dwi Putra:** Data Curation, Statistical Analysis.

## Data Availability

Mendeley Data2D Data for Training Artificial Neural Networks in Measuring Depression Levels (Original data).
